# Combined Strategy of Wound Healing Using Thermo-Sensitive PNIPAAm Hydrogel and CS/PVA Membranes: Development and In-Vivo Evaluation

**DOI:** 10.3390/polym14122454

**Published:** 2022-06-16

**Authors:** Yan Chu, Shuo Chai, Fei Li, Cuiyan Han, Xiaoyu Sui, Tingting Liu

**Affiliations:** College of Pharmacy, Qiqihar Medical University, Qiqihar 161006, China; 2019220124@stu.qmu.edu.cn (Y.C.); 2020220115@stu.qmu.edu.cn (S.C.); 2021220133@stu.qmu.edu.cn (F.L.); hancy1001@126.com (C.H.)

**Keywords:** wound dressing, electrostatic spinning, PNIPAAm hydrogel, nanofiber membrane, CuS nanoparticles

## Abstract

Past studies have shown that the hot spring effect can promote wound healing. Mild thermal stimulation and metal ions can promote angiogenesis. In this study, the hot spring effect was simulated by thermosensitive PNIPAAm hydrogel loaded with copper sulfide nanoparticles. Heat stimulation could be generated through near-infrared irradiation, and copper ions solution could be pulsed. On the other hand, the CS/PVA nanofiber membrane was attached to the bottom of the hydrogel to simulate the extracellular matrix structure, thus improving the wound healing ability. The CS/PVA nanofiber membrane was prepared by electrospinning, and the appropriate prescription and process parameters were determined. The nanofiber membrane has uniform pore size, good water absorption and permeability. The poor mechanical properties of PNIPAAm hydrogel were improved by adding inorganic clay. The temperature of the hydrogel loaded with CuS nanoparticles reached 40 °C under near-infrared light irradiation for 20 min, and the release rate of Cu^2+^ reached 26.89%. The wound-healing rate of the rats in the combined application group reached 79.17% at 13 days, demonstrating superior results over the other control groups. Histological analyses show improved inflammatory response at the healed wound area. These results indicate that this combined application approach represents a promising wound treatment strategy.

## 1. Introduction

Skin trauma represents an important global public health problem. With the development of civilization, humans have created many means of treating wounds, including herbs, potions, ointments and surgical sutures. Hot spring treatment is a traditional and effective therapy method of wound healing in many countries. The French and Japanese have a long history of treating wounds with hot spring water [1,2]. The therapeutic effects of hot springs are mainly related to two aspects. The first is the mild thermal stimulation of a 30–45 °C hot water bath. Studies have shown that a hot bath can increase the blood vessel density of granulation tissue and promote wound healing. On the other hand, hot springs contain many minerals, including sulfur, sodium, copper, sulfur, iron, calcium, and silicic acid, which bear anti-inflammatory properties and promote wound healing [3]. Inspired by the hot spring treatment, we propose the use of a polymer hydrogel system to simulate the hot spring effect. The key issue in designing the hydrogel system centers on methods to facilitate the sufficient release of bioactive ions from the hydrogel and creation of mild thermal stimulation for the wound.

Near-infrared photothermal conversion materials have been widely used in medical treatment. Under the local irradiation of near-infrared light with a certain wavelength, the photothermal material can rapidly switch from the ground state to the excited state, and then dissipate to the external environment in the form of heat [4]. Semiconductor photothermal conversion materials are a new type of near-infrared-induced photothermal conversion materials, which are simple in preparation, high in absorption coefficient and stable in photothermal performance. This type of material mainly includes metal chalcogenides and metal oxides [5]. Among them, copper sulfide nanoparticles (CuS NPs) have attracted much attention due to their good biocompatibility, low toxicity and low cost [6,7]. CuS NPs show near-infrared absorption at 700–1000 nm, and the absorption does not depend on the dielectric constant of the surrounding medium, and the effect of nanoparticle morphology is negligible [8,9]. Previous studies reported that CuS NPs can be efficiently metabolized, and the clearance rate through hepatobiliary and renal excretion is greater than 90% after injection for one month [10]. In addition, studies have shown that copper sulfide nanoparticles in aqueous solution can release copper ions, which can stimulate the secretion of vascular endothelial growth factor (VEGF), thereby promoting angiogenesis [11]. Poly(N-isopropylacrylamide) (PNIPAAm) is currently the most widely used temperature-responsive polymer in the medical field, and is generally designed as a hydrogel for application [12]. PNIPAAm has a lower critical solution temperature (LCST), in which it induces reversible dehydration from a helical to spherical change. Therefore, the PNIPAAm hydrogel exhibits controlled drug release properties [13,14,15]. At present, some studies have combined photothermal conversion materials with PNIPAAm hydrogels to modulate drug release by light irradiation. Moreover, depending on the thermal effect of the photothermal conversion material, the temperature of the hydrogel can also be maintained within a certain range [16,17]. Therefore, using near-infrared light to irradiate the PNIPAAm hydrogel loaded with copper sulfide nanoparticles can not only rapidly release the copper ion solution, but also heat the environment around the hydrogel to simulate a hot spring bath.

Although the use of photothermal hydrogels that release bioactive ions can simulate a hot spring bath, there are two shortcomings. Firstly, the bioactive ion solution rapidly released by photothermal can easily leak from the wound. Secondly, the hydrogel surfaces do not effectively mimic the structure of the extracellular matrix (ECM). The extracellular matrix of human skin tissue is a highly fibrous and porous three-dimensional network that supports cell growth and promotes tissue formation [18,19]. The application of electrospun nanofibers has received increasing attention in biomedicine. With high specific surface area, high porosity, and good interconnectivity, nanofibers have great potential to mimic skin extracellular matrix in morphology and composition. Studies have shown that nanofibers can support keratinocyte adhesion and spreading, and smooth fiber surfaces can provide more contact points for cell adhesion, making them suitable for use as wound dressings [20,21,22]. In addition, nanofibers composed of polymers have swelling properties and can absorb a certain volume of water. At present, there are many studies on blended nanofibers based on chitosan (CS), mainly due to the excellent biocompatibility and antibacterial effect of chitosan [23,24].

Based on the above considerations, we designed a novel strategy of combining copper sulfide nanoparticles/PNIPAAm hydrogel with CS/PVA nanofiber membrane for wound healing. In this scheme, PNIPAAm hydrogel loaded with copper sulfide nanoparticles is mainly used to simulate the hot spring effect. The release of copper ions is controlled by near-infrared light irradiation, and mild thermal stimulation is provided to the wound area. The CS/PVA nanofiber membrane was used to prevent the loss of copper ion solution at the wound, and to play a role in inhibiting bacteria and promoting cell proliferation. We investigated the formulation, process parameters, and properties of the nanofibrous membrane and PNIPAAm hydrogel, respectively, and evaluated the therapeutic effect of wound dressing on wound healing in rats.

## 2. Materials and Methods

### 2.1. Materials

NIPAAm, N,N′-methylenebis (acrylamide) (BIS), tetramethylenediamine (TEMED) and ammonium persulphate (APS) were procured from Sigma Aldrich Co., Ltd. (Shanghai, China). CuCl_2_ and Na_2_S were procured from Shanghai Macklin Biology Technology Co., Ltd. (Shanghai, China). Clay (Mg_5.35_Li_0.66_)Si_8_O_20_(OH)_4_(Na_0.66_) was purchased from Aoyuan new material technology Co., Ltd. (Hebei, China). PVA and CS (degree of deacetylation ≥ 95%) were purchased from Sinopharm Chemical Reagent Co. Ltd. (Beijing, China). Pure water was prepared using a Milli-Q water purification system (Millipore, Bedford, MA, USA).

### 2.2. Solutions Preparation

First, 1 wt% CS solution was prepared by dissolving some CS powdered in 5% acetic acid at room temperature under magnetic stirring for 12 h. Then, 4 g PVA was dissolved in distilled water at 90 °C under stirring for 2 h, and after cooling to room temperature, a PVA solution (8 wt%) was mixed with a CS solution (1 wt%) at a weight ratio of (CS/PVA) 1:1. Thereafter, the mixture was continuously stirred at room temperature for 6 h. The finally obtained blend solution was prepared for electrospinning [25]. 

### 2.3. Electrospinning

The electrospinning apparatus consisted of a high voltage power with 25 kV, a syringe pump and a collector covered with aluminum foil (Beijing Ucalery Industry Technology Development Co., Ltd., Beijing, China). The CS/PVA solution was loaded in a 5 mL syringe equipped with a 21 gauge needle for the electrospinning process. The applied voltage ranged between 11–15 kV. The distance between the tip and the collector was fixed at 15–18 cm and the feeding rate of the electrospinning solution was 0.1–0.3 mm/min. Finally, the resulting CS/PVA nanofibrous membrane was dried at room temperature for 24 h for use.

### 2.4. Synthesis of CuS Nanoparticles

The synthesis of CuS NPs was carried out as a facile route as follows. Briefly, 100 μL CuCl_2_ solution (300 mmol/L) was added to 10 mL PVP solution (100 mg/mL), then 30 μL Na_2_S solution (1 mol/L) was added, room temperature magnetic stirring for 5 min, then transferred to 90 °C water bath and continued magnetic stirring for 15 min, and in this process, the color of the reaction solution gradually changed from light blue to light yellow, and finally to dark green. The CuS NPs were formed [26]. The morphology of the CuS NPs was observed by transmission electron microscopy (TEM) performed on a Hitachi HT7700 (Japan). CuS NPs were dispersed with pure water and their mean size were characterized using a laser particle-size analyzer (Nano ZS90 Malvern Instruments, Worcestershire, UK) [27]. The concentrations of Cu^2+^ were measured using UV–Vis spectrophotometry. The experiment is performed based on the Fenton reaction principle, where the presence of Cu^2+^ in the assay solution catalyzes the decomposition of hydrogen peroxide to produce hydroxyl radicals. The latter oxidizes the colorless 3,3′,5,5′-tetramethylbenzidine (TMB) in the solution to the yellow TMB^2+^, which can be used for quantitative analysis Cu^2+^ by measuring the absorbance of TMB^2+^ at 452 nm.

### 2.5. Preparation of PNIPAAm Hydrogels

Hydrogels were prepared using initial solutions consisting of monomer (NIPAAm), cross-linker (Clay and BIS), solvent (H_2_O), initiator (APS), and catalyst (TEMED). The synthetic procedure of hydrogel formation is simple. First, a transparent aqueous solution consisting of water (10 mL), Clay and BIS (0.038 g), and NIPAAm (1.04 g) was prepared. Next, the catalyst (TEMED, 40 µL) and subsequently the aqueous solution of initiator (APS 0.038 g in H_2_O 3 mL) were added to the former solution with stirring at iced-water temperature. Then, free-radical polymerization was allowed to proceed in a water bath at 0 °C for 20 h. Throughout the experiments, oxygen was excluded from the system. Hydrogel was purified prior to use by immersion in an excess of water at 2 °C for 48 h, changing the water several times.

### 2.6. Characterization of Electrospun Membrane

#### 2.6.1. Water Absorption Test of the CS/PVA Membrane

The nanofiber membrane with a certain size was cut, the recording mass *W*_1_ was weighed, and then immersed in distilled water for 1 h. After being removed, filter paper was used to absorb the surface moisture of the dry film, and *W*_2_ was weighed again to calculate the water absorption rate of the sample. The formula was as follows:

(1) $$\mathrm{Water}\;\mathrm{Absorption}\;(\%)=\frac{W_{1}-W_{2}}{W_{1}}\times 100\%$$

where *W*_1_ represents the weight of initially nanofiber membrane, W_2_ represents the weight of nanofiber membrane in distilled water for 1 h, respectively. Three samples were tested for each group.

#### 2.6.2. Air Permeability Test of the CS/PVA Membrane

A 6 mL volume of distilled water was added into the penicillin bottle, the bottle mouth area of penicillin was marked as S, and then the bottle mouth was sealed with nanofiber membrane, and mass *W*_1_ was weighed [28]. Then, the samples were placed into an incubator at 37 °C for culture for 24 h, and *W*_2_ was weighed again after being removed, to calculate the air permeability of the samples, with the following formula:

(2) $$\mathrm{Air}\;\mathrm{Permeability}\;(\%)=\frac{W_{1}-W_{2}}{S}\times 100\%$$

where *W*_1_ represents the weight of initially nanofiber membrane, *W*_2_ represents the weight of nanofiber membrane at 37 °C for 24 h, the bottle mouth area of penicillin was marked as *S*. Three samples were tested for each group.

### 2.7. Scanning Electron Microscopy (SEM)

The surface morphology of the samples was observed using an S-4300 scanning electron microscope (Hitachi, Tokyo, Japan).

### 2.8. Characterization of Hydrogel

#### 2.8.1. Swelling Ratio Measurement

The swelling ratios of the hydrogels were measured under different temperatures ranging from 20 °C to 40 °C. The samples were immersed in the water for a certain time, then the swollen hydrogels were extracted, wiped with filter paper to remove excess water from the surface, and weighed [29,30]. The swelling ratio (SR) was calculated as follows:

(3) $$\mathrm{Swelling}\;\mathrm{Ratio}\;(\%)=\frac{M_{t}-M_{0}}{M_{0}}\times 100\%$$

where *M*_0_ and *M_t_* represent the weight of the hydrogels and the weight of the swollen hydrogel under different conditions, respectively. Three samples were tested for each group.

#### 2.8.2. Deswelling Kinetics Analysis

The hydrogel swollen to equilibrium at 25 °C was quickly transferred into hot water of 45 °C. At this time, the hydrogel shrunk and lost water. The hydrogel was extracted at regular intervals and weighed for recording. The Water Retention (WR) rate of the hydrogel was calculated as follows [31]:

(4) $$\mathrm{Water}\;\mathrm{Retention}\;(\%)=\frac{M_{t}-M_{0}}{M_{e}-M_{0}}\times 100\%$$

where *M*_0_ and *M_t_* represent the weight of initial hydrogels and the weight of the swollen hydrogel at time t, *M_e_* represents the weight of hydrogel at 25 °C, respectively. Three samples were tested for each group.

#### 2.8.3. Water-Retaining Test

The hydrogel samples after reaching swelling balance with similar size and equal mass were stored at room temperature. The samples were weighed at a predetermined time until the weight of the sample was constant, and the water retention rate SR (%) of the samples were calculated according to a formula [32]:

(5) $$\mathrm{Swelling}\;\mathrm{Ratio}\;(\%)=\frac{M_{t}-M_{0}}{M_{e}-M_{0}}\times 100\%$$

where *M*_0_ and *M_t_* represent the weight of initially hydrogels and the weight of the hydrogel at time t, *M_e_* represents the weight of hydrogel at 25 °C, respectively. Three samples were tested for each group.

### 2.9. Measurement of Cu^2+^ Release from Hydrogels

The completely-dried hydrogel was thoroughly soaked in a beaker containing 1.5 mL of CuS nanoparticles for 48 h at room temperature until the hydrogel swelled and balanced, and the hydrogel was then extracted [33,34]. The hydrogel was washed with deionized water to remove residual CuS nanoparticles on the surface of the hydrogel. The hydrogel was placed in a dry Petri dish and irradiated with near-infrared light (808 nm, 1 W/cm^2^). The temperature change was recorded during the irradiation. The hydrogel in the Petri dish was removed and washed with 1 mL of deionized water, and the content of copper ions in the release solution was determined by UV–Vis spectrophotometry [35,36].

### 2.10. Determination of Reabsorption Rate after Light-Controlled Drug Release

The hydrogel loaded with CuS nanoparticles was taken out, weighed and recorded with mass of *M*_0_, placed on a 2 cm × 2 cm nanofiber membrane in a dry Petri dish. After near-infrared light (808 nm, 1 W/cm^2^) was applied for 20 min, the weighed hydrogel was recorded with mass of *M_t_*, the hydrogel was placed at room temperature and restored below the LCST, and the weight of the hydrogel was recorded as *M*_1_. The reabsorption rate and the release rate of the samples were calculated according to the formula:

(6) $$\mathrm{Release}\;\mathrm{rate}\;(\%)=\frac{M_{0}-M_{t}}{M_{0}}\times 100\%$$

(7) $$\mathrm{Reabsorption}\;\mathrm{rate}\;(\%)\frac{M_{1}-M_{t}}{M_{1}}\times 100\%$$

### 2.11. Wound Healing Assay

The wound healing efficacy of the prepared dressing was evaluated using a full-thickness excisional wound model. The animal studies were carried out on adult male and female Wistar rats (weighing 200–250 g). All animal experiments were performed in accordance with protocols evaluated and approved by the Ethical Committee of Qiqihar Medical University. Chloral hydrate (0.04 mL/100 g body weight) was administrated through intraperitoneal injection to induce general anesthesia. A full-thickness excisional wound (1.0 cm × 1.0 cm) was induced on the back skin of rats [37,38]. Afterward, the animals were randomly divided into six groups (six rats in each group), including PNIPAAm-PVA/CS-CuS with illumination, PVA/CS hydrogels-PVA/CS nanofiber membrane-CuS with illumination, PNIPAAm-PVA/CS-CuS without illumination, PVA/CS nanofiber membrane, PNIPAAm-CuS hydrogels, and the negative control group (treated with sterile gauze). An elastic adhesive bandage was used to fix the wound dressing. The dressing was changed daily, and then illuminated with near-infrared light for 30 min. The wound area was then measured with a ruler and recorded by taking pictures.

### 2.12. Histological Analysis

The histopathology studies were used to evaluate the healing efficacy of the treatments. After 5, 9 and 14 days of treatment, the animals were euthanized, the skin tissue harvested, and fixed in buffered formalin (10%, pH = 7.26) for 48 h. Fixed tissues were processed, embedded in paraffin, sectioned into 5 μm slices, and stained with hematoxylin and eosin (H&E) stains. Finally, the epithelialization, angiogenesis, fibroplasia, and granulation tissue formation were evaluated using light microscopy (Olympus BX51; Olympus, Tokyo, Japan) equipped with a digital camera (Olympus, Tokyo, Japan).

### 2.13. Data Analysis

All statistical evaluations were conducted with SPSS for Windows, version 17.0.0 (SPSS Inc., Chicago, IL, USA). A value of *p* < 0.05 was considered to be statistically significant.

## 3. Results

### 3.1. The Performance Evaluation of Hydrogel

Due to the nature of high-molecular-weight polymers and the plasticizing effect of water molecules, PNIPAAm hydrogels are generally weak in mechanical strength and fragile. Clay was used as a crosslinking agent to improve the mechanical strength of the hydrogel [39]. Clay is a colloidal, water-containing layered aluminosilicate, most of which is naturally formed and belongs to the class of phyllosilicates. According to the type of ion exchange, clay can be divided into cationic clay and anionic clay. Laponite is a cationic clay, and hydrotalcite is an anionic clay. Laponite is a synthetic hectorite with the molecular formula (Mg_5.35_Li_0.66_)Si_8_O_20_(OH)_4_(Na_0.66_). It can significantly improve the mechanical properties of hydrogels given that clay can form multiple hydrogen bonds with polymer molecular chains, effectively increasing the crosslinking density of hydrogels [40]. Next, the swelling rate and other properties of the hydrogel were used as evaluation indicators to determine the amount of different crosslinking agent.

#### 3.1.1. Swelling Ratio Measurement

**Figure 1:**
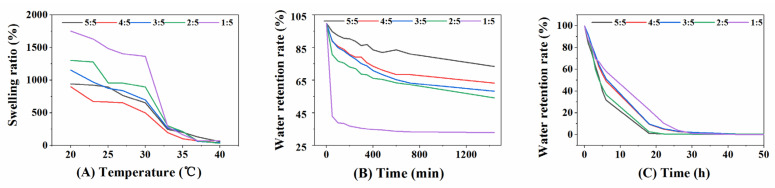
(**A**) Swelling ratios of the PNIPAAm-BIS-Clay hydrogels as a function of temperature, (**B**) Deswelling kinetics of the PNIPAAm-BIS-Clay hydrogels at 45 °C, (**C**) water retention of the PNIPAAm-BIS-Clay hydrogels.

The equilibrium swelling degree of the hydrogel was an important parameter. The change in the equilibrium swelling degree represents a macroscopic manifestation of the conformational changes of the macromolecular chain [41]. The equilibrium swelling degree of the hydrogel was used to reveal the relationship of the microscopic interaction state and the polymer chain conformation. For wound dressing hydrogels, the swelling properties are related to the absorption of wound exudate. Figure 1A shows the relationship between the swelling degree of the PNIPAAm hydrogel in deionized water and temperature. Figure 1A demonstrates that the swelling rate of the hydrogel decreases with increasing temperature, and the swelling rate decreases significantly at 30–33 ℃. As the clay content increased, the slope of the swelling rate curve becomes larger, and the temperature sensitivity becomes more obvious. The hydrogel with BIS:clay = 1:5 was the most sensitive to temperature. The ability of clay to enhance the swelling properties of hydrogels may be due to entanglement or physical cross-linking between clay particles and hydrogels. In addition, the hydrogen bonding between clay molecules and water molecules enhances the ability of the hydrogel to absorb water [42].

#### 3.1.2. Deswelling Kinetics Analysis

Deswelling performance is closely related to the regulation of drug release. Figure 1B shows that the deswelling rate of the PNIPAAm-BIS-clay hydrogel has a decreasing trend over time. As the clay content in the hydrogel increases, the degree of deswelling of the hydrogel also increases. Among them, the hydrogel with BIS:clay = 1:5 demonstrated a faster deswelling rate. This may be due to the increase in the number of pores and water channels inside the hydrogel as the clay content increases, which facilitates the outward diffusion of water molecules [43]. 

#### 3.1.3. Water-Retaining Test

Maintaining a moist environment assists wound healing, and water retention is an important indicator for evaluating the moisturizing ability of a wound dressing [44]. Hydrogel, with a high water-retention rate, was more conducive to its application. According to Figure 1C, the water retention rate of the PNIPAAm-BIS-clay hydrogel was ranked as BIS:clay = 1:5 > 4:5 > 3:5 > 2:5 > 5:5. Among them, the average water retention rate reached 58.18% after the hydrogel with BIS:clay = 1:5 lost water at room temperature (T = 25 °C, H = 23%) for 6 h and continued at room temperature (T = 25 °C, H = 23%). Water was lost for 24 h, and the average water retention rate was 3.98%.

**Figure 2:**
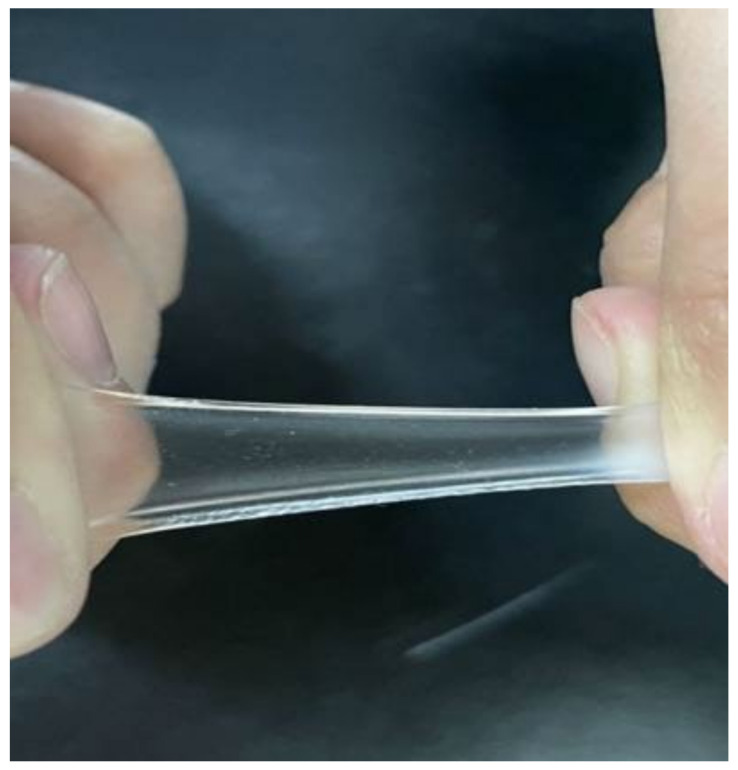
Photograph demonstrating the stretching effect of hydrogel.

According to the above information, the dosage of BIS:clay = 1:5 was used for the next research. According to Figure 2, the hydrogel prepared under this dosage demonstrated better stretchability.

### 3.2. Influence of Electrospinning Solution Parameters on the Fiber Membrane Quality

Electrospinning devices usually consist of syringe pumps, capillary needles (spinnerets), high-voltage power supplies, and metal collectors. In the electrospinning process, a high voltage was generated to create a charged jet of polymer solution, which was directed to the collector by electrostatic force, thereby creating an interconnected fiber membrane. The characteristics of electrospun membranes depend on the properties of the precursor solution (conductivity, surface tension, viscosity, and solvent selection), process variables (flow rate, voltage, and distance between the spinneret and collector), and environmental conditions (temperature and humidity). The control of these specific parameters has a direct effect on the average diameter and arrangement of the nanofibers produced.

#### 3.2.1. Influence of Spinning Solution Concentration on Spinnability

First, 1 wt%, 2 wt%, and 3 wt% CS solution and 8 wt% and 11 wt% PVA solutions were mixed at a mass ratio of 5/5 and stirred at room temperature for 4 h to obtain spinning solutions of different concentrations, denoted A1, A2, A3, A4, A5, and A6, and the spinning effect was observed.

Table 1 shows the spinning effect of different concentrations of spinning solution. When the PVA concentration was 11 wt%, it had no spinnability after mixing with 3 wt% CS, but it had spinnability after mixing with 1 wt% and 2 wt% CS, and a small amount of liquid was dropped during spinning. When the PVA concentration was 8 wt%, it had spinnability after mixing with 3 wt%, 2 wt%, and 1 wt% CS, and as the concentration of the CS solution decreased, the spinning effect improved. Increasing the CS concentration reduced the spinnability of the solution, and a high concentration of PVA rendered the solution too viscous, resulting in reduced jet stability [45]. Therefore, we will blend 1 wt% CS and 8 wt% PVA in different proportions for follow-up studies.

#### 3.2.2. Influence of CS/PVA Mixed-Solution Ratio on Spinnability

The 1 wt% CS solution and 8 wt% PVA solution were mixed at mass ratios of 8/2, 7/3, 6/4, 5/5, 4/6, and 2/8 to obtain different blending ratios of the spinning solution, denoted as A, B, C, D, E, F, and G for spinning.

**Figure 3:**
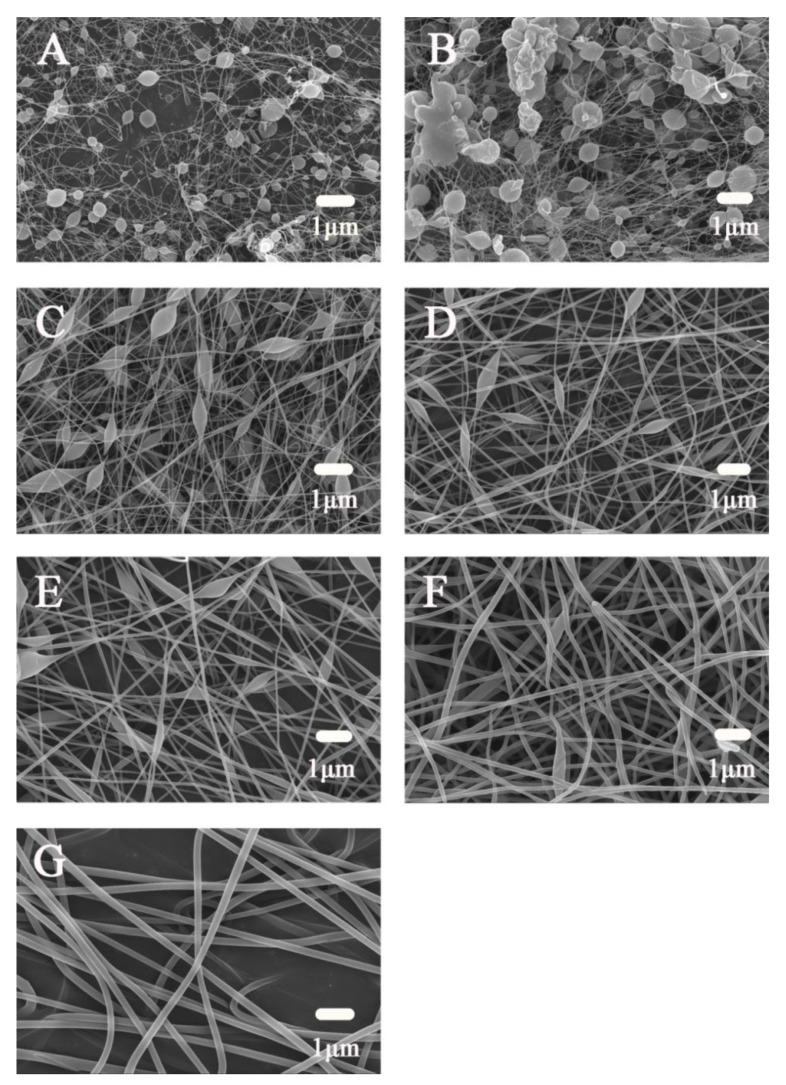
Effects of CS and PVA blend ratio on fiber morphology ((**A**) CS:PVA = 8:2; (**B**) CS:PVA = 7:3; (**C**) CS:PVA = 6:4; (**D**) CS:PVA = 5:5; (**E**) CS:PVA = 4:6; (**F**) CS:PVA = 3:7; (**G**) CS:PVA = 2:8).

Table 2 and Figure 3 show the spinning conditions of the spinning solutions with different blending ratios and scanning electron microscopy (SEM) images of the prepared nanofiber membranes, respectively. Among them, the fiber morphology of G was best: the diameter was relatively uniform, the distribution was concentrated, and there was no spindle. The spinnability of F in the electrospinning process was poor, and spindles appeared. This was because the movement of molecules at higher viscosity makes it difficult for the jet to deform and split at the electrospinning nozzle. When the proportion of CS was increased, a large number of beads and spindles appeared in the fiber morphology. This might be because in the CS/PVA mixture, when the content of CS reaches a certain level, PVA continuously separates the phase distribution in the form of fibers. CS was deposited in the form of beads and could not be fiberized.

SEM showed that the nanofiber membrane of G was relatively uniform in diameter, concentrated in distribution, and absent of spindles. On the other hand, because CS was a bacteriostatic component, to ensure its content, the F state of the nanofiber membrane was also considered better, so the two ratios of F and G were selected for the next investigation.

### 3.3. Influence of Electrospinning Process Parameters on the Properties of Spinning Films

Modern wound healing theory suggests that wound dressings should be able to maintain a suitably moist environment. The air permeability of the nanofiber membrane can ensure that the water of the hydrogel layer can pass through and maintain a moist environment. In addition, the growth of blood vessels and the proliferation of cells in the wound area depend on the supply of oxygen, and the air permeability of the fibrous membrane is closely related to the supply of oxygen. On the other hand, good penetration performance can ensure that copper ions reach the wound site through the spinning membrane, realizing rapid drug release, and improving bioavailability. Therefore, the best process parameters were selected based on the air permeability and water absorption as the evaluation index [46].

#### 3.3.1. Spinning Voltage’s Influence

In this experiment, 1 wt% CS solution and 8 wt% PVA solution were mixed at mass ratios of 2/8 and 3/7 to obtain a spinning solution. The prepared spinning solution was placed into a 5 mL syringe, 21-gauge needle was installed, the spinning voltage (11 kV, 13 kV, 15 kV) under the conditions of advancing speed of 0.1 mm/min was changed, the receiving distance was 15 cm, and receiving time of 5 h.

**Figure 4:**
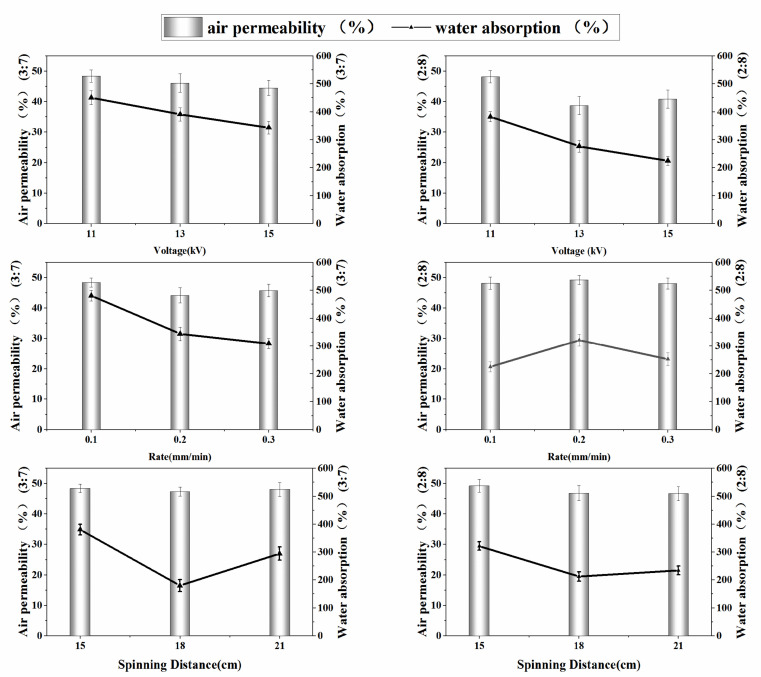
Effect of CS and PVA spinning voltage, advancing speed and spinning distance on water absorption and air permeability.

Figure 4 shows the water absorption and air permeability of nanofiber membranes spun under different spinning voltages. As shown, when the voltage was 11 kV, the water absorption and air permeability of nanofiber membranes with mass ratios of CS to PVA of 2/8 and 3/7 were the highest. As the spinning voltage increases, the water absorption and air permeability of the nanofiber membrane with a mass ratio of CS to PVA of 3/7 are reduced, while the nanofiber membrane with a mass ratio of CS to PVA of 2/8 has a voltage of 2/8. Air permeability was poorest at 13kV. The reason for the above results may be that during the spinning process, the increase in voltage increases the speed of the polymer jet, which leads to a decrease in its stability, thus affecting the fiber membrane performance.

From the experimental results, 11 kV was concluded as the optimal voltage parameter, thus a spinning voltage of 11 kV was selected for the next parameter study.

#### 3.3.2. Advancing Speed’s Influence

The 1 wt% CS solution and 8 wt% PVA solution were mixed at mass ratios of 2/8 and 3/7 to obtain a spinning solution. Speed (0.1 mm/min, 0.2 mm/min, and 0.3 mm/min) was changed under the conditions of a spinning voltage of 11 kV, a receiving distance of 15 cm, and a receiving time of 5 h to study its effect on nanofiber membrane performance. 

Figure 4 shows the water absorption and air permeability of nanofiber membranes spun at different advancing speeds. When the advancing speed is 0.1 mm/min, the water absorption and air permeability of the nanofiber membrane with a mass ratio of CS to PVA of 3/7 are the highest, and as the advancing speed increases, both water absorption and air permeability decrease to varying degrees. The nanofiber membrane with a mass ratio of CS to PVA of 2/8 reached the highest at a speed of 0.2 mm/min. In the spinning process, it was observed that too small or too high of an advancing speed caused decreased stability of the jet. Appropriate increase in the speed of progress would be beneficial to improve the efficiency of spinning.

Based on the above results, we chose 0.1 mm/min and 0.2 mm/min as the advancing speed of the blended liquid spinning with mass ratios of CS to PVA of 3/7 and 2/8, respectively.

#### 3.3.3. Receiving Distance’s Influence

The solution was obtained by mixing 1 wt% CS and 8 wt% PVA at mass ratios of 3/7 and 2/8, with a spinning voltage of 11 kV, advancing speed of 0.1 mm/min and 0.2 mm/min, and spinning time of 5 h. Under these conditions, the receiving distance (15 cm, 18 cm, 21 cm) was changed to collect the spinning fiber.

Figure 4 shows the water absorption and air permeability of nanofiber membranes spun at different receiving distances. It can be seen from the figure that when the receiving distance was 15 cm, the water absorption and air permeability of nanofiber membranes with mass ratios of CS to PVA of 2/8 and 3/7 were optimal. Generally, a shorter receiving distance means a stronger electrostatic field and faster polymer jet deposition on the collecting plate. As the receiving distance increases, the water absorption and air permeability of nanofiber membranes with mass ratios of CS to PVA of 3/7 and 2/8 both show different degrees of decrease, which might be due to a longer fiber-receiving distance. The electrostatic field strength reduced movement speed of the polymer jet, thereby prolonging receiving time, resulting in a significant decrease in the degree of fiber collection. According to the experimental results, we determined that the appropriate receiving distance was 15 cm.

The process conditions were determined from the measurement results: PVA:CS = 7:3, spinning voltage of 11 kV, advancing speed of 0.1 mm/min, spinning distance of 15 cm; and PVA:CS = 8:2, spinning voltage of 11 kV, advancing speed of 0.2 mm/min, and receiving distance of 15 cm.

### 3.4. Determination of CS/PVA Ratio

**Figure 5:**
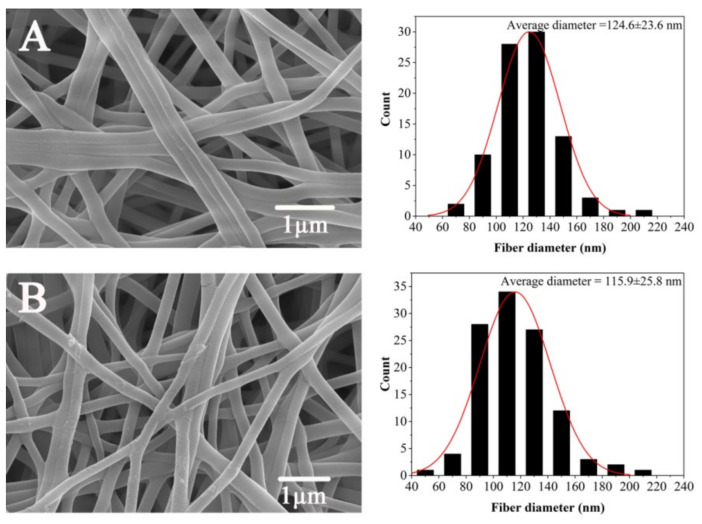
Scanning electron microscopy of nanofiber membrane ((**A**) CS:PVA = 3:7; (**B**) CS:PVA = 2:8).

Figure 5 shows the SEM images of nanofiber membranes prepared with spinning solutions with mass ratios of CS to PVA of 3/7 and 2/8 under the respective suitable spinning parameters. The fiber with a mass ratio of CS to PVA of 2/8 exhibits better morphology, more uniform diameter and good porosity. It can be seen from the histogram in Figure 5A that the average diameter of the nanofibers is 115.9 + 25.8 nm. While the fiber with a mass ratio of CS to PVA of 3/7 is poor, the average diameter of the fiber is 124.6 + 23.6 nm. The increase in PVA content not only improves the properties of the spinning solution but also improves the spinnability of CS. Therefore, we used a blend with a CS/PVA mass ratio of 2/8. The nanofiber membrane was prepared under previously described spinning parameters.

### 3.5. The Morphology of CuS Nanoparticles

**Figure 6:**
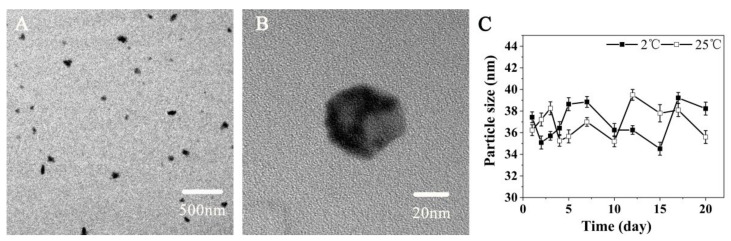
TEM images of CuS nanoparticles and particle size stability of CuS nanoparticles in difference time. (**A,B**) TEM images of CuS nanoparticles, (**C**) The particle size stability of CuS nanoparticles in difference time.

Figure 6B shows the polyhedron shape of CuS nanoparticles under a transmission electron microscope, with good dispersibility and an average particle size of approximately 40 nm. As can be seen in Figure 6A, some nanoparticles exist in the form of dimers and trimers. The CuS nanoparticles were placed at 2 °C and 25 °C, and particle size was measured regularly. According to statistics (Figure 6C), there was no significant difference in the size change of CuS nanoparticles within 20 days, and all were stable in the range of 35–39 nm, indicating good particle size stability.

### 3.6. The Photothermal Effect of CuS-Loaded Hydrogel

**Figure 7:**
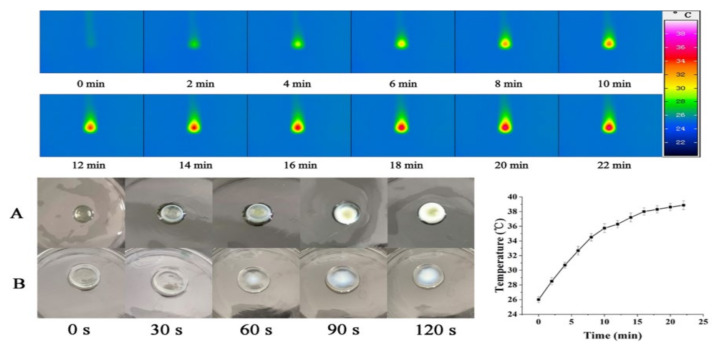
Temperature curve of CuS nanoparticles under 808 nm near-infrared light and photothermal performance. (**A**) The hydrogel loaded with CuS nanoparticles, (**B**) The hydrogel loaded without CuS nanoparticles.

As shown in Figure 7, under near-infrared light irradiation (808 nm) at a power of 1 W/cm^2^, the heating rate was approximately 1 °C/min. After 22 min of irradiation, the temperature rose from 20 °C to 40 °C and without any change [47]. The temperature rose gradually throughout the heating process. The hydrogel loaded with CuS NPs cannot only be observed with an obvious temperature change in the thermal infrared imager (Figure 7), but can also be seen as a transparent hydrogel due to the photothermal conversion of CuS nanoparticles, the colloid temperature being higher than the LCST (Lower Critical Solution Temperature), the weakening of the hydrogen bonding between amide groups and water molecules, and the strengthened hydrophobic interaction between polymer chains, leading to sharp contraction. Macroscopically, the hydrogel visually transforms from transparent to white (Figure 7A). When the temperature is lower than the LCST, the strong hydrogen bonding between amide groups and water molecules in PNIPAAm chain increases hydrophilicity of the polymer chain, and the hydrogel will change again from white to transparent [48]. The temperature change of the hydrogel without CuS nanoparticles was not obvious (Figure 7B).

### 3.7. Determination of Cu^2+^ Concentration in Hydrogel under Near-Infrared Light Irradiation

**Figure 8:**
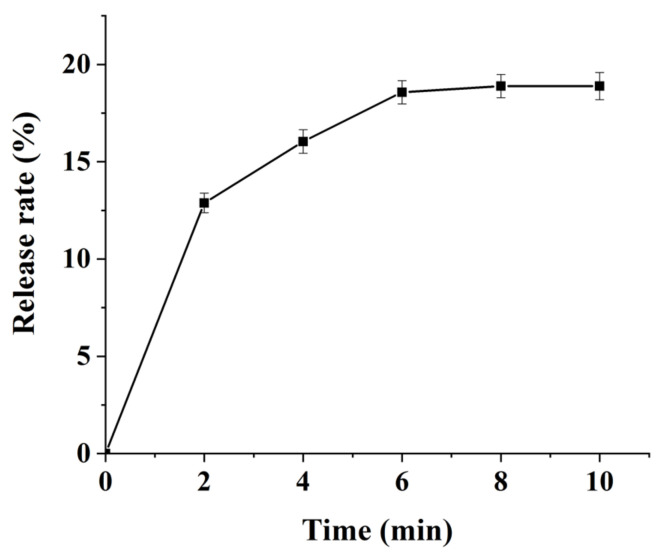
The Cu^2+^ release curve of hydrogel loaded with CuS NPs under near-infrared light irradiation.

Figure 8 shows the release curve of Cu^2+^ from hydrogels under near-infrared light irradiation. Within 0–6 min, the release rate of Cu^2+^ is continuous. After 6 min, the released copper ion concentration begins to plateau. At the 10th minute, the release rate was 26.89%. The reason for this may be that the high temperature of the near infrared (808 nm) irradiation site causes the hydrogel to undergo a phase change and the drug to be released. In approximately 6 min, the hydrogel completes the phase change, and the volume change ceases indicating that most of the drugs have been released [49].

### 3.8. Determination of Reabsorption Rate after Light-Controlled Pulse Drug Release

**Figure 9:**
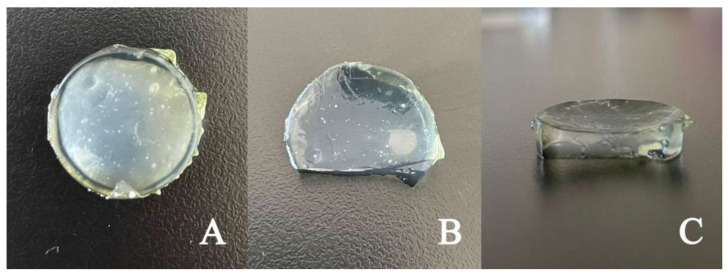
The state of combined application of hydrogels and nanofiber membranes. (**A**) Fit state after complete wetting (The nanofiber membrane is in the lower layer), (**B**) fit state after complete wetting (The nanofiber membrane is in the upper layer), (**C**) cross-section angle (The nanofiber membrane is in the upper layer).

In this part, we measured the mass change of the combined hydrogel and nanofiber membrane system after controlled release by light in order to investigate whether the hydrogel would demonstrate reabsorption after pulse release of aqueous solution. The mass of the samples after irradiation with near-infrared light (808 nm, 1 W/cm^2^) for 20 min and gradual recovery to below LCST at room temperature data are shown in Table 3. It can be seen from the data that the photo-controlled pulse release rate of hydrogel is about 30%. The resorption rate is about 4.4% when the temperature returns to below the LCST. Through the reabsorption experiment, it was found that the released part of the aqueous solution was almost completely absorbed by the nanofiber membrane in the lower layer of the hydrogel. After absorption, the nanofiber membrane swelled, showing a transparent state and a smooth surface (Figure 9). The results show that the combined application of nanofiber membrane and hydrogel can not only prevent the outflow of the pulsed Cu^2+^ solution, but also maintains the wet state of the nanofiber membrane. It can be speculated that if the system is applied to a wound, it can keep the wound surface moist, and allow full Cu^2+^ contact with the wound. This will promote epidermal migration and angiogenesis and prevent wound drying and cell death.

### 3.9. In Vivo Wound Healing

**Figure 10:**
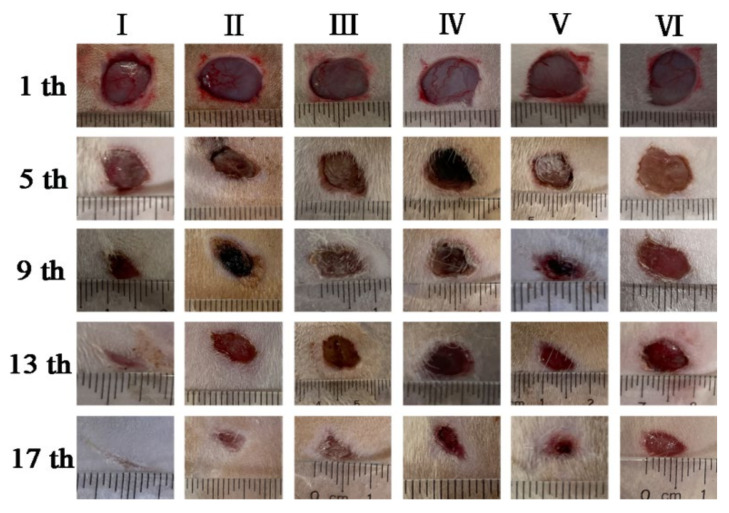
Macroscopic photographs of wounds at 1st, 5th, 9th, 13th and 17th day after full-thickness ((**I**) PNIPAAm-PVA/CS-CuS with illumination, (**II**) PVA/CS hydrogels-PVA/CS-CuS with illumination; (**III**) PNIPAAm-PVA/CS-CuS without illumination; (**IV**) PVA/CS nanofiber membrane; (**V**) PNIPAAm-CuS hydrogels; (**VI**) treated with sterile gauze).

Wound healing is shown in Figure 10. In the early stage of wound healing, there was no significant difference in the healing between groups given that the wound model created was roughly circular with a diameter of 1 cm, and its recovery was based on the wounded skin [50]. On the 5th day after modelling, there was a small amount of inflammatory exudate on the wound surface, and the wounds in group VI showed the moistest appearance. On the 9th day, the wound healing rate of rats in Group I was significantly accelerated, and there were almost no symptoms of infection, and the wound area was smaller compared to other control groups. On the one hand, this difference may be related to the fact that drug-loaded nanofibers can increase migration of keratinocytes. On the other hand, copper sulfide can also promote angiogenesis at the wound site. Loading it into a temperature-sensitive hydrogel can increase its bioavailability compared with ordinary hydrogels, thereby accelerating wound healing. The healing of Group V was also better, and there were scabs in the wounds of groups II, III, and IV. The wounds of group VI showed obvious signs of infection, including abscess and inflammation. On the 13th day, healing was basically completed in group I, followed by group V, and groups II, III, and IV also accelerated significantly. There was still clear redness, swelling, and inflammation in group VI. On the 17th day after modelling, Group I demonstrated an almost complete recovery, followed next by the healing of groups II and V [51].

### 3.10. Wound Closure Evaluation Index

The wound diameters of each group were determined, and the percentage of healing was calculated. The results are shown in Table 4.

The healing rate of group I was faster than others on the 5th day, and the healing rates of group II and group V were similar. By the 13th day, the healing rate of group I reached 79.17%, followed by group V. At the 17th day, the healing rate of group I reached 89.33%, which was greater than that of group VI (gauze group) at 50%.

The general linear model was used to compare the healing rates among different groups. Statistical analysis showed that there were significant differences (*p* < 0.05) between group I and group II, and between group I and group III. The result indicated that the fast release of copper ions generated by the temperature-sensitive hydrogel under irradiation with near-infrared light can promote wound healing [52]. The photothermal effect induced by near-infrared light irradiation also can accelerate wound healing to a certain extent. The comparison between group I, group VI, and group V shows that the combined application strategy healed faster than using PNIPAAm hydrogel or CS/PVA spinning membrane alone.

### 3.11. Histological Analysis

**Figure 11:**
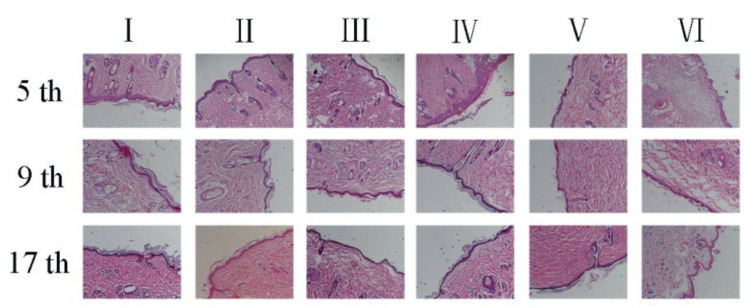
Hematoxylin and eosin (H&E) stained microscopic sections of healed incisions in rats. ((**I**) PNIPAAm-PVA/CS-CuS with illumination, (**II**) PVA/CS hydrogels-PVA/CS-CuS with illumination; (**III**) PNIPAAm-PVA/CS-CuS without illumination; (**IV**) PVA/CS nanofiber membrane; (**V**) PNIPAAm-CuS hydrogels; (**VI**) treated with sterile gauze).

Figure 11 shows the H&E stained tissue analysis of rat wound repair. Wound healing is a complex biological process involving various cells and tissue components, including regeneration of various tissues, epithelialization of wounds, and granulation tissue formation. Through the H&E staining method, pathological changes in skin wound tissue can be observed at each time point.

After five days of wound healing, the epidermal layer was repaired in the treatment group. The epidermal layers of groups IV and V was very thin, while the epidermal layer of group VI had not yet formed, and a large number of inflammatory cells infiltrated and aggregated on the wound surface. Group IV had the fewest inflammatory cells, indicating an antibacterial effect of the CS, and Group I exhibited a large number of new blood vessels (ovular, mostly in the wound) [53].

After nine days of wound repair, hair follicle structures were observed in each group. Group VI still contained a small number of inflammatory cells and a very thin epidermal structure; in the experimental group, granulation tissue and fibroblasts can be seen, which have completed epithelialization and are rich in new blood vessels.

On the 16th day of wound repair, the epidermal structure of group VI was still incomplete. In the treatment group, a large number of collagen fibers were generated in the wound tissue, and an epidermal structure was formed. The structure of each layer was regular and orderly, and the surface structure was close to normal skin. The granulation tissue had formed and grown into the wound. The model site was filled with fibroblasts. The wound skin tissue is similar to normal tissue.

## 4. Conclusions

In this study, hydrogel prepared by the mixed crosslinking agent BIS:clay = 1:5 demonstrated better stretchability. CuS nanoparticles with photothermal properties were loaded into the hydrogel. Under 808 nm near-infrared light irradiation with a power of 1 W/cm^2^, the hot spring environment was simulated. The composite hydrogel undergoes a phase change and releases Cu^2+^ at a release rate of 26.89%. The nanofiber membrane exhibits uniform diameter, a loose form and porosity between fibers, and has good water absorption and air permeability. The wound model showed that the combined application of PNIPAAm hydrogel and nanofiber membrane could enhance wound healing. The results of the histological analyses found that the combined application of hydrogel and nanofiber membrane in comparison with other groups improved dermis development and inflammatory response. Therefore, the combined application of PNIPAAm hydrogel and nanofiber membrane demonstrates potential for use as an effective wound dressing in biomedical applications. In addition, blending of the PNIPAAM hydrogel-CuS nanoparticle films of different thicknesses with the CS/PVA membranes of different composition and porosity can open a new area of more applicable wound dressing materials in future research.

## Figures and Tables

**Table 1:** Spinning effect of CS/PVA spinning solution with different concentrations.

Number	The Concentration Ratio of CS	The Concentration Ratio of PVA	Conditions (Voltage, Range, Velocity)	Result
A1	3 wt%	11 wt%	10 kV, 16 cm, 0.3 mm/min	without spinnability
A2	2 wt%	11 wt%	11 kV, 15 cm, 0.3 mm/min	poor spinnability, instability
A3	1 wt%	11 wt%	11 kV, 15 cm, 0.3 mm/min	poor spinnability, instability
A4	3 wt%	8 wt%	11 kV, 15 cm, 0.3 mm/min	good spinnability
A5	2 wt%	8 wt%	11 kV, 15 cm, 0.1 mm/min	good spinnability
A6	1 wt%	8 wt%	11 kV, 15 cm, 0.2 mm/min	good spinnability, stability

**Table 2:** Spinning effect of CS/PVA spinning solution with different blending ratios.

Number	The Concentration Ratio of CS	The Concentration Ratio of PVA	Conditions (Voltage, Range, Velocity)	Result
A	8	2	11 kV, 15 cm, 0.1 mm/min	Unable to form fibers
B	7	3	11 kV, 15 cm, 0.1 mm/min	Unable to form fibers
C	6	4	11 kV, 15 cm, 0.1 mm/min	Continuous fibers with a large number of spindles
D	5	5	11 kV, 15 cm, 0.1 mm/min	Continuous fibers with a large number of spindles
E	4	6	11 kV, 15 cm, 0.1 mm/min	Continuous fibers with a small number of spindles
F	3	7	11 kV, 15 cm, 0.1 mm/min	Continuous fibers with a small number of spindles
G	2	8	11 kV, 15 cm, 0.1 mm/min	Continuous fibers without spindles

**Table 3:** Reabsorption rate and release rate of hydrogel after pulse release.

No.	M_0_ (g)	M_t_ (g)	M_1_ (g)	Reabsorption Rate (%)	Release Rate (%)
1	0.9116	0.6087	0.6369	4.6	33.2
2	0.9387	0.6210	0.6511	4.8	30.6
3	0.9148	0.6565	0.6821	3.9	28.2

**Table 4:** Results of percent wound healing on different days, values represent the mean ± SD, *n* = 6.

Days	Healing Percentage (%)					
	Ⅰ	Ⅱ	Ⅲ	Ⅳ	Ⅴ	Ⅵ
5	33.33 ± 1.66	25.00 ± 1.12	16.67 ± 0.91	16.67 ± 0.88	25.00 ± 1.12	12.50 ± 0.80
9	50.00 ± 2.42	37.50 ± 1.87	20.83 ± 1.00	37.50 ± 1.94	37.50 ± 1.85	33.33 ± 1.66
13	79.17 ± 3.15	45.83 ± 2.22	33.33 ± 1.65	41.67 ± 2.23	62.50 ± 3.05	37.50 ± 1.75
17	89.33 ± 4.12	70.67 ± 3.31	56.25 ± 2.17	62.50 ± 3.25	79.17 ± 3.85	50.00 ± 2.50

## Data Availability

The data presented in this study are available on request from the corresponding author.
